# Heart Images on Food Labels: A Health Claim or Not?

**DOI:** 10.3390/foods10030643

**Published:** 2021-03-18

**Authors:** Krista Miklavec, Maša Hribar, Anita Kušar, Igor Pravst

**Affiliations:** 1 Nutrition and Public Health Research Group, Nutrition Institute, Tržaška cesta 40, SI-1000 Ljubljana, Slovenia; krista.miklavec@nutris.org (K.M.); masa.hribar@nutris.org (M.H.); anita.kusar@nutris.org (A.K.); 2 Biotechnical Faculty, University of Ljubljana, Jamnikarjeva 101, SI-1000 Ljubljana, Slovenia; 3 VIST–Higher School of Applied Sciences, Gerbičeva cesta 51A, SI-1000 Ljubljana, Slovenia

**Keywords:** health claim, brand name, word association, conjoint analysis, heart image, front-of-package labelling

## Abstract

Health claims on food labels are used by food manufacturers to inform consumers about the health effects of a product, and such claims can have notable effects on consumer preferences. According to regulatory definitions, health claims can be either worded or presented as images, but it is not clear under which conditions an image on a food label should be considered a health claim. This question has important practical implications, as the use of health claims is strictly regulated. The objective of this study was to determine how commonly images of the heart are used on food labels, and to investigate consumers’ perceptions of products labelled with heart images, using different degrees of health relationships. Both a food supply study (N = 10,573 foods) and experiments with consumers (N = 1000) were performed in Slovenia. The use of heart imagery on food products was very common (9%). The consumer study was conducted using a web panel. Structure of the study population was comparable with Slovenian adult population (18–65 years), according to gender and age. The questionnaire was split into conjoint analysis with constructed elements, a choice-based task with real-life elements and a consumers’ association task. The experiments showed that a heart image as part of the brand name itself—without an additional (worded) health claim—did not cause most consumers to relate it to health. However, consumers tended to strongly relate an image of the heart as part of a brand with health benefits, where the image was accompanied by a worded health claim or if the heart image was designed specifically to imply health benefits. We can conclude that the use of heart images was very common on food products, but references to health were less common. Without a health-related context, heart images could not be considered as a health claim.

## 1. Introduction

Food labels can, on the one hand, support consumers in making informed choices, while on the other hand, they can be used as a strong marketing tool. To ensure that the information provided on food labels is relevant for consumers, food labelling is carefully regulated. While there are considerable regulatory differences between different countries [1], a key objective of such legal rules is to ensure that consumers are sufficiently informed about the properties and composition of specific food products, supporting informed choices. Another important goal of food labelling regulations is to prevent practices that could mislead consumers. In the European Union (EU), mandatory food labelling is regulated by Reg. No 1169/2011 concerning the provision of food information to consumers, while additional regulations cover non-mandatory information that can also be provided on food labels, such as nutritional information and health claims [2].

Nutritional information and health claims are typical examples of non-mandatory information commonly provided on food labels [3]. As their use can affect consumers’ choice of food products and therefore, directly influence nutrient intakes, such claims are commonly regulated more carefully. For example, in the EU, the wordings of health claims and their conditions of use must be subject to pre-approval and inclusion on a list of permitted health claims [4]. Pictures on food labels and trademarks or brand names are no exception when these could be interpreted as nutritional or health claims. Each image or symbol that could be related to health need to be accompanied with an authorised nutritional or health claim, in line with the regulatory conditions of use [2]. However, the EU health-claims regulation provides an exemption—a trademark or brand name that existed before 1 January 2005 can remain in use until 19 January 2022; thereafter, all foods will need to fully comply with the regulatory requirements.

The end of this transition period is bringing considerable challenges in the area of food labelling in Europe, affecting not only local food producers but also market-leading international food brands. The origin of the problem lies in the very wide definition of a health claim. According to EU regulations, a health claim is “any message or representation (including pictorial, graphic or symbolic representation, in any form), which states, suggests or implies that a relationship exists between a food category, a food or one of its constituents and health” [2]. According to this, health-related images can be interpreted as health claims, and even if these are part of decades-old trademarks, such elements could become illegal on food labels after 2022, if not complying with the regulation. For example, if a trademark is implying heart health, it should be accompanied with authorised specific health claim, referring do heart health. However, there are no specific authoritative recommendations (beyond the regulatory definition of a health claim) that support deciding if a specific use of an image is considered a health claim. Food authorities in different EU countries can, therefore, interpret these rules very differently; the same or a very similar image on a label can be interpreted as a health claim in one country but not a health claim in another country.

While several studies from around the globe have reported the prevalence of nutritional and health claims on food products [3,5,6,7,8,9,10,11,12,13,14,15,16], these are mostly focused on worded claims. However, a CLYMBOL (Role of health-related claims and symbols in consumer behaviour) study conducted in five EU countries reported the use of trademarks/brand names that could be interpreted as health claims on 17% of analysed prepacked foods [3], highlighting that regulatory changes could affect many products in the food supply. An example of a very common element on food labels is an image/symbol of the heart.

Several studies have shown that consumers struggle to understand nutritional and health claims on foods [17,18,19,20,21] and often interpret claims differently from regulators [22]. Consumers make their purchase decisions based on a number of factors [23]. Some may identify themselves with the brand, so the brands can influence consumers’ buying decisions [24,25]. The logo related to the brand contributes to consumers’ decisions on whether they like the brand, especially since there are many similar products on the market and the brand could be the main differentiator [26]. A change in the brands’ logo could affect those consumers and their buying decisions [27].

**Figure 1:**
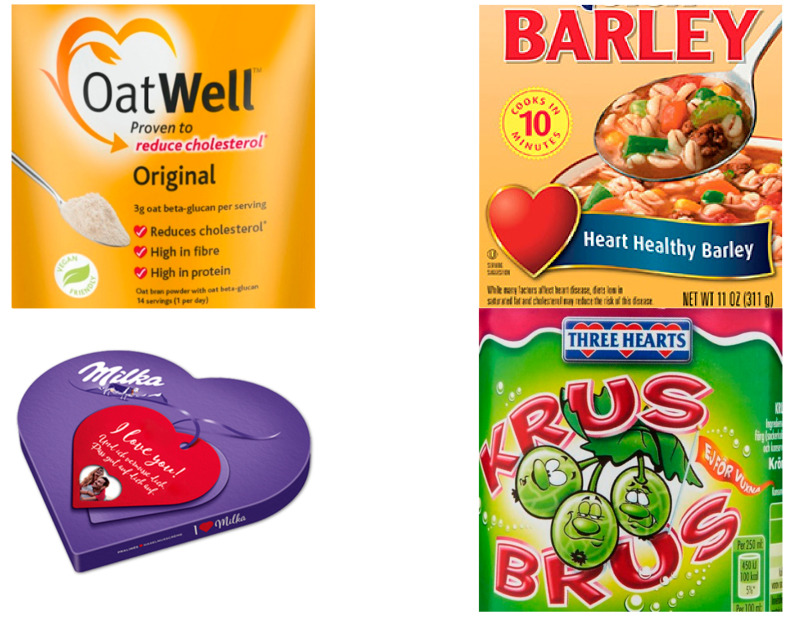
Examples of food labels with heart images in the international food supply (see Acknowledgements section for details).

There has been very limited research into when an image or trademark on a food label becomes a health claim. One reason could be the subjectivity of evaluation, as some images or trademarks do not directly imply connections to health, but consumers could still relate them to it and vice versa. For example, food labelling can contain a heart symbol (♥) as part of an “I ♥ food” message (without any reference to health) or the heart symbol can be part of a clear health claim (e.g., “For healthy ♥ heart”). However, in many cases, the use of a heart image/symbol on a food label falls somewhere between these two borderline situations, and the decision on whether the symbol presents a health claim is much more complex (see some examples provided in Figure 1). Klepacz et al. used a false-recollection method to detect implicit inferences about products’ health benefits [28]. They conducted series of experiments, in which participants saw fictional product packages, accompanied by written claims. While some packages contained an image implying a health-related function, others did not. After participants were exposed to these packages and claims, researchers tested their memory for seen and unseen claims. Study results showed that images alone can indeed present a health claim, as they lead people to conclusions about health benefits, even if not supported with additional elements. Authors proposed their method for measuring the potential of specific images to (mis)lead. However, this memory-based method is appropriate for newly developed images, and not for existing, well-recognised brands.

In this study, we focused on the use of heart images on food labels. Our goals were to determine how commonly images of the heart are used on food labels, and to investigate consumers’ perceptions of products labelled with heart images, using different degrees of health relationships (a heart image alone, and a heart in combination with worded health claims). The food supply study was conducted in Slovenia, and the consumer experiments were conducted using a web panel; the tested materials were developed using real and constructed food-labelling elements. The food-labelling elements were constructed using images of hearts with/without specific/general health claims. With the permission of the brand owners, some experiments were conducted using the real trademark (and its elements) of Radenska^®^ mineral water, which is composed of an image of three red hearts. This is one of the largest premium mineral water products in the Adriatic region [29]. The brand has existed for more than 150 years, and the company has used three heart images in the trademark since 1936, highlighting the three different water springs.

## 2. Methodology

The research consisted of two parts. First, we looked at how often heart images appeared on food labels, and secondly, we performed a consumer study to learn about consumers’ perceptions of products labelled with heart images.

### 2.1. Food Supply Study: Penetration of Heart Images/Symbols on Food Labels

The food supply study was conducted on images of food products in the Slovenian food supply. Data collection was described previously [30]; in brief, it was performed in the supermarkets of three major retailers (Spar, Hofer and Mercator) in Slovenia, using the Global Food Monitoring Initiative (GFMI) protocol [31]. The selected retailers cover the majority of the national market. As part of the study, the European Article Number (EAN) barcodes were scanned to accelerate the database’s formation and avoid duplicate entries. All prepacked foods in the food stores were systematically photographed (in agreement with the store owners), and food labelling data were recorded in an online Composition and Labelling Information System (CLAS) database. Out of 10,674 recorded products in the CLAS 2015 database, 10,573 remained in the database for further analyses (about 100 products were removed due to poor picture quality or not meeting inclusion criteria, i.e., multipacks containing multiple, different food products). For this specific study, photographs of food products were checked for the use of any images/symbols of the heart. We determined the penetration of images/symbols of the heart on food labels. This was done by two experienced researchers with nutrition backgrounds. To provide interrater reliability, both researchers evaluated a subsample of foods. Good agreement [32] was observed with minor discrepancies, particularly related with missed background stylised silhouettes in the shape of s heart. Discrepancies were resolved to ensure consistency of further data collection. A decision was taken that also vague silhouettes of a heart counted as images of the heart for this study. Additionally, two food categories were selected for additional examination. Among food categories with the highest absolute number of foods with a heart on the front-of-package (FOP, bread and bakery products; meat and meat products; dairy; cereal and cereal products; n > 100 per category), dairy and cereal and cereal products also had highest number of foods with a heart as part of the brand (n > 40 per category). These two categories were, therefore, selected for further examination. We investigated the contexts in which the heart symbols tended to appear on food labels (context of use, i.e., a heart-shaped food package, a cut-out window, a promotion or endorsement, illustrative, as part of the product name or branding, a reference to the ingredient quality, the country of origin, or a health or nutrition claim). Considering the sample size of this sub-sample (N = 230), it was manageable for two researchers to conduct this coding together; all open issues were discussed within the research team on case-by-case basis to ensure consistency of data collection.

### 2.2. Consumer Study

The consumer study was conducted using a GfK web panel with 1000 people aged 18–65, via an online questionnaire in October 2016. The sample was representative of the Slovenian population according to age and gender. Data collection was conducted by GfK Slovenia.

The questionnaire was split into different sections: (1) conjoint analysis with constructed elements, (2) a choice-based task with real-life elements, (3) consumers’ associations with an example of a brand containing health images and (4) sociodemographic and life-style characteristics (Table 1).

#### 2.2.1. Conjoint Analysis

**Figure 2:**
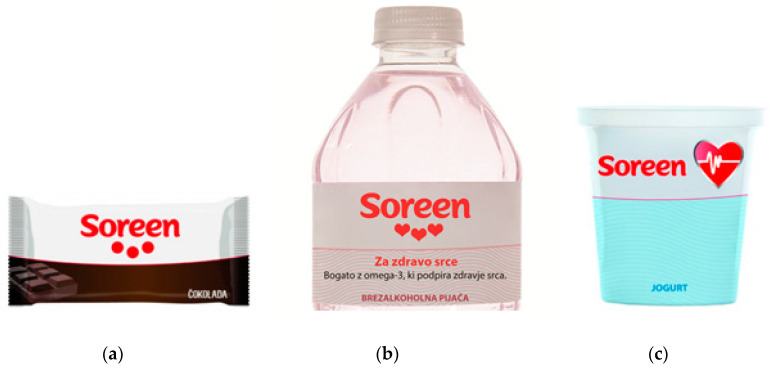
Examples of stimuli used in the conjoint analyses task: (**a**) Chocolate with a neutral brand (•••); (**b**) soft drink with three hearts (♥♥♥); (**c**) yoghurt with health-implying heart image (♥).

Conjoint analysis (CA) is a method used to estimate the importance consumers allocate to different predefined attributes [33]. It has been employed in many studies to show consumers’ preferences towards certain foods or food label attributes [34,35,36,37,38,39,40,41,42,43]. Our design considered three attributes—(a) the type of product, (b) the brand name and (c) claims; each consisted of three attribute levels (see Table 2 and examples of stimuli in Figure 2). For this purpose, we constructed the brand name “Soreen”, which cannot be found on the Slovenian market, so the consumers were not familiar with it. That is how we avoided bias that could have resulted if using a recognised brand in the sorting task. This imaginary brand name was then used to construct different types of visual stimuli with expressed health benefits. As consumers perceive the healthiness of different types of foods very differently, the experiment used three different food matrices (type of product: chocolate and soft drinks as an example of less healthy food, and yoghurt from the healthier food category). For the claim attribute, we used health claims (general health claim alone, or accompanied with a specific health claim), or no claim at all. Considering that the study was investigating heart images, tested health claims were referring to heart health. We used a simple general health claim ‘For a healthy heart’ (in Slovenian: ‘Za zdravo srce’). According to EU regulations, general health claims shall be accompanied with related specific claims. There are two authorised function health claims in the EU register of health claims [44], specifically referring to heart health—one for omega-3 fatty acids (for foods enabling a daily intake of 250 mg omega-3 fatty acids in form of eicosapentaenoic acid and/or docosahexaenoic acid), and one for vitamin thiamine (for foods which meet requirements for use of the nutrition claim ‘source of thiamine’). We decided to use the one referring to omega-3 fatty acids in our study. Full wording of tested specific health claim was ‘For a healthy heart-rich in omega-3, which supports a healthy heart’ (in Slovenian: ‘Za zdravo srce—bogato z omega-3, ki podpira zdravje srca.’).

The full factorial design comprised 27 (3 × 3 × 3) different profiles; to minimize them into a manageable format for the participants to evaluate, we used an orthogonal fractional factorial design that enabled all the attributes to be equally represented while maintaining statistical reliability. The final number of profiles used was nine; each was individually presented to the participants. They needed to decide how the consumption of the product shown would impact their health on a scale of 1 to 7 (1 = unfavourable impact and 7 = favourable impact). In line with CA methodology [33], the relative importance of individual attributes and the part-worth utilities of attribute levels were calculated.

In an extension of the CA experiment, the participants were asked about the health impacts of additional soft-drink products (the same question as in the CA experiment, but with additional stimulus images). For the stimuli already included in the CA experiment, previously collected data were used. The dataset of responses for the soft drink profiles therefore included all three types of brand name (••• neutral, ♥ one heart, and ♥♥♥ three hearts) and three types of claim (no claim, a general claim, and a specific health claim) on a scale of 1 to 7 (1 = unfavourable impact and 7 = favourable impact). Altogether, each participant evaluated all nine stimulus variations for soft drinks.

#### 2.2.2. Choice-Based Task with Real-Life Elements

We presented the participants with a choice-based task involving real-life brand names to investigate if heart images alone affected the perceived health benefits of the products. We used water as a food matrix, enabling us to test the use of the image of three hearts as part of the well-established Radenska^®^ brand name (see the Introduction). We constructed stimuli for three typical bottled waters from Slovenian market: (1) Oda; (2) Jana; and (3a/b) Radenska, where the Radenska stimuli included (3a) or did not include (3b) heart images (Figure S1). The participants were asked to compare the health benefits of the tested product with those of tap water (note that in Slovenia, tap water is drinkable and considered to be of high quality), from 1 (less beneficial than tap water) through 5 (equally beneficial) to 9 (more beneficial than tap water). The exact formulation of the question was (translated): “It is well established that water is essential for the normal functioning of body and therefore beneficial to health. Please rate on a scale from 1 to 9 how beneficial this water is in comparison with regular tap water.” Such a rating was used because water is generally considered to be healthy, so the scale used in the CA experiment (1 = unfavourable impact; 7 = favourable impact) would have poor sensitivity on the positive side of the scale. In the experiment, each participant was asked about three stimulus images: either 1 + 2 + 3a or 1 + 2 + 3b (randomised selection; ratio, 1:1). The stimuli (1) and (2) were actually meant as an exercise for rating the stimuli 3a/b, where we were interested in how the presence/absence of the image of three hearts affected the product rating. The difference between the groups was assessed using *t*-tests (paired *t*-tests for comparing 1/3 and 2/3, and independent *t*-tests for comparing 3a/3b).

#### 2.2.3. Word-Association Task

**Figure 3:**
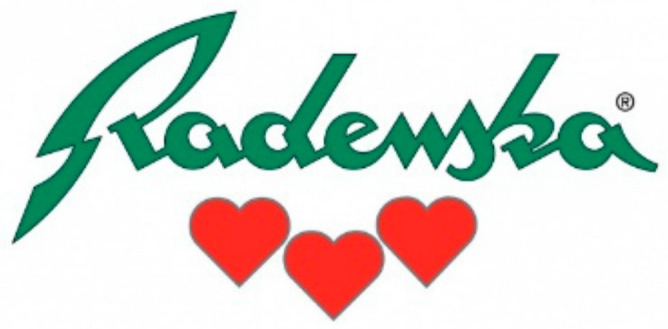
Radenska^®^ brand name, used in the word-association task.

After the choice-based task with real-life elements, the Radenska brand was shown to all the participants in its original form. They were asked to write down the first thing that came to mind when they saw the image of the logo (Figure 3). Word association is a simple and quick qualitative method regularly used by psychologists and sociologists [45] and has been useful for extracting information from consumers about a brand [46]. The words stated in such a task are more spontaneous than closed questionnaires, which may produce more biased results [47], even though its greatest disadvantage is the complexity of data analysis, along with the subjectivity in how the responses are interpreted [46]. However, word-association tasks with symbols/pictures have recently been used in many different studies [38,43,48,49,50]. The word-association task was included in the last part of the questionnaire to avoid influence on the previous tasks, where the use of heart images was investigated.

### 2.3. Data Processing and Statistical Analyses

In the Food Supply Study (Section 2.1) data were processed using Microsoft SQL Server Management Studio 13.0, Microsoft Analysis Services Client Tools 13.0, Microsoft Data Access Components (MDAC) 10.0 and the CLAS (Nutrition Institute, Ljubljana, Slovenia). Descriptive statistical analysis was done using Microsoft Excel 16.0 (Redmond, Washington, DC, USA).

In a consumer study and conjoint analysis (Section 2.2 and Section 2.3) the data collection tool was prepared using the SPSS Data Collection Software. The relative importance of individual attributes and the part-worth utilities of attribute levels were calculated using IBM^®^ SPSS (13.0) (New York, NY, USA), while other analyses were carried out in Microsoft^®^ Excel (16.0) (Redmond, Washington, DC, USA). In the choice-based task with real-life elements (Section 2.2.2), group differences were investigated using the *t*-test. Differences were considered significant at *p* < 0.05.

## 3. Results and Discussion

### 3.1. Use of Heart Images on Food Labels

We investigated the penetration of the appearance of heart images on food labels in the Slovenian food supply, primary focusing on the front-of-package (FOP) labelling. Altogether, 10,573 food packages were examined for the use of heart images, silhouettes, etc. If the packaging was in the shape of a heart (e.g., some chocolate boxes), this was also considered as a FOP use of the heart symbol. Altogether, 9% of the items carried the heart symbol FOP, while 3% carried it as part of the product’s brand name. The highest percentages of items with the heart symbol were found among foods for specific dietary use (51%). This was not a surprise, as this regulated food category also contains food for special medical purposes and total diet replacement for weight control. Quite a high penetration of the heart symbol was also found in eggs (29%), meat and meat products (16%), edible oils and emulsions (15%), bread and bakery products (12%), convenience food (11%), and cereal and cereal products (10%). Heart images were present on 7% of beverages, mostly waters (33%) and soft drinks (8%). The results for all the examined food categories are presented in Table 3 (see also Table S1 for results according to specific sub-categories). To the best of our knowledge, this is first study to investigate this topic; therefore, we are not able to make any direct comparisons with other countries. However, some previous research examined the use of brand names that could be interpreted as health claims; it was shown that about 17% of the prepacked foods analysed in five EU countries carried brand names/trademarks that could be perceived as health claims [51]. However, as already mentioned, judging if a brand name/trademark could be perceived as a health claim is quite challenging, as images can be perceived differently among different consumers and researchers. Therefore, we first identified any use of any heart image/symbol on food labels, without consideration of the context. To gain further insights into the use of heart images on food labels, we took a closer look at the food labels within the two selected food categories, to investigate the contexts in which heart images appear on food labels. To make this task manageable, we focused into two food categories (Cereal and cereal products, and Dairy products– see Methodology Section 2.1). We determined that heart images are mainly used as part of the brand name, endorsement, and nutritional or health claims within cereal and cereal products. Within dairy, heart images are most commonly presented as part of the country of origin and brand name.

### 3.2. Consumer Study

**Figure 4:**
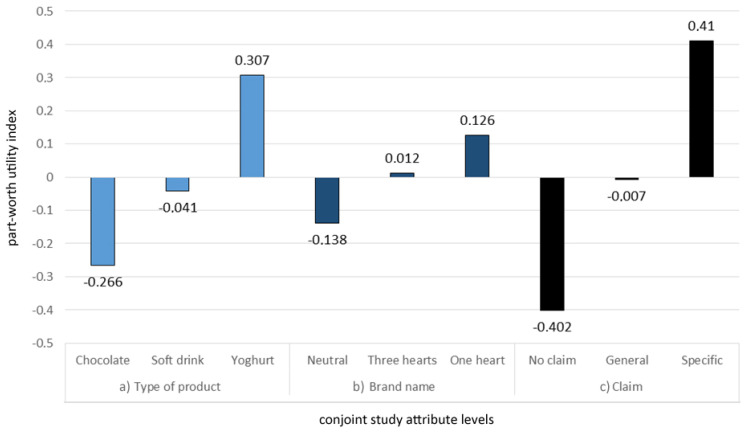
Results of conjoint analyses with part-worth utilities of study attribute levels: (**a**) Type of product: Soft drink/Yoghurt/Chocolate; (**b**) Brand name: Neutral/One heart/Three hearts; and (**c**) Claim: No claim/General health claim “For a healthy heart”/Specific health claim: “For a healthy heart—rich in omega-3, which supports a healthy heart.” (N = 1000, Slovenia).

While our goal was to investigate consumers’ perceptions of products labelled with brands, constructed with different degrees of health relationships (Figure 2), the experimental design also included different food categories and worded references to health, as these were expected to be important factors affecting consumers’ perceptions. Indeed, the results of the CA study showed that type of product (food category) was the product attribute with the highest relative importance (42%), followed by claims (34%) and the brand name (24%). As expected, the part-worth utilities (Figure 4) were the highest for yoghurt (0.307) and lowest for chocolate (−0.266), while soft drinks had a slightly negative part-worth utility (−0.041). We tested three types of brands; one without any heart images (•••; Figure 2a), one with an image of three hearts without any reference to health (♥♥♥; Figure 2b), and one with an image of a heart, with an implied heart–health relationship (♥, Figure 2c). It should be noted, again, that the participants were asked about the expected health benefits of the product. A neutral brand name (•••) had a negative part-worth utility (−0.138), while the other two types of brand names had positive part-worth utilities (0.012 for a brand name with one heart (♥) and 0.126 for a brand name with three hearts (♥♥♥)). Additionally, a positive part-worth utility (0.410) was observed for the specific health claim “For a healthy heart—rich in omega-3, which supports a healthy heart”; neutral (−0.007), for general health claim “For a healthy heart”; and negative (−0.402), when no claim was present. To summarise the CA results, regarding the impact of the use of heart images on health perceptions, the type of product was of great importance for the participants, as was the visual presentation of the brand name and, in particular, how clear the connection of the image with health effects was. Clear effects were observed when a heart image with an implied health relationship was additionally supported with an explanatory health claim. On the other hand, a brand name with an image of three red hearts (without an additional health association) did not have a relevant effect on participants’ evaluations if not accompanied by a worded health claim.

We reported in a previous study that the wording accompanying symbols has an important effect on consumers’ choices, especially if there is a strong implication for health; on the other hand, when a claim did not accompany the symbol, negative part-worth utilities were observed [49]. Ballco, et al. [52] also reported that consumers prefer products that carry nutrition and health claims. Moreover, research showed that brand name itself did not affect consumers’ responses, while the package shape and product type influenced the perceived healthfulness of the product [53]. These results show that a product should be considered as a whole, not according to one element only.

In addition to the CA study, for one selected food category (soft drinks), we also analysed participants’ responses to all possible variations of the brand name/claim. The participants were again asked about the expected health impact, using a Likert scale (1—unfavourable; 7—favourable impact). The results in Table 4 show that the difference between the means is not as big among different brands as among the accompanying claims. This shows that while the images on brands can affect consumer expectations regarding health effects, much more pronounced effects occurred when the brands were accompanied with worded health claims. For example, a neutral brand (•••) with a specific health claim was rated notably higher than a heart health-implying brand (♥), with average means of 4.7 ± 1.4 vs. 4.1 ± 1.4, respectively. It should be noted that the brand “Soreen” was used in our study because this word has no specific meaning in the Slovenian language and is not present in any brand name in the Slovenian food supply. Therefore, the study participants did not have any pre-study experiences or expectations related with such a brand. However, this could have also affected the study results. Velasco Vizcaíno and Velasco [54] reported that if consumers are unfamiliar with the brand, they pay more attention to FOP labels. Considering this, it might be the case that the observed effects of the symbols (•••, ♥ and ♥♥♥) and accompanying health claims were higher than they would have been had we tested a familiar brand name. On the other hand, if a brand has been systemically communicated to consumers with reference to specific health effects, such a brand could have a much stronger effect on consumers’ expectations regarding health effects, even if such effects are not communicated on the food labelling. Additionally, Klepacz et al. showed, with memory-based methods, that images on food labels can lead people to conclusions about health benefits if such images have previously been connected with health expectations [28].

Next, the participants completed a choice-based task with real-life brand names familiar to consumers. We used brands of bottled water typical in the Slovenian market: (1) Oda, (2) Jana, and (3a/b) Radenska. The expectations of health benefits were measured on a Likert scale from 1 to 9, using tap water as a reference (5). Water labels were constructed using these real-life brands, of which only one (Radenska^®^) contains a picture of three hearts (♥♥♥) (see Figure 3). The experiment was conducted in such a way that half of the participants were exposed to the original Radenska brand name (with three hearts; Figure S1/3a), while half saw this brand name without the image of the three hearts (3b). The mineral water brand Radenska (♥♥♥; 3a) received the highest rating (mean, 4.7 ± 2.2), followed by the other two tested products (4.0 ± 2.2 and 3.9 ± 2.1 for (2) and (1), respectively). This was in line with our expectations, considering that Radenska is a market-leading mineral water brand with more than 150 years of tradition in the Adriatic region [29]. However, the main goal of this experiment was not to study brand-to-brand differences but to check the influence of the image of three hearts as part of the real-life brand on consumers. Comparisons of the ratings of the label with (3a) and without (3b) the image of three hearts showed negligible and non-significant differences, with mean ratings of 4.8 ± 2.2 and 4.7 ± 2.2, respectively (*p* = 0.48, independent *t*-test). On the other hand, the differences in the ratings of different brands ((1)/(2) vs. (3a)) were not only much greater but also statistically significant (*p* < 0.001, paired *t*-test).

It is interesting that the mean ratings were below 5 for all the tested bottled waters, which was reference for tap water. This can be explained by the fact that in Slovenia, tap water is high in quality and drinkable across the country. To gain further insight into this, we separately checked the ratings for participants who reported the regular consumption of bottled water (at least one per day; N = 121). As expected, the mean ratings for bottled water (in comparison to tap water) were notably higher; the differences between the brands were in line with the trends observed in the full sample, but less pronounced (mean ratings, 5.8 ± 2.1, 5.4 ± 2.1 and 5.3 ± 2.1 for (3), (2) and (1), respectively). A considerable proportion of the study participants reported tap water as healthier, in comparison with bottled water. This might be related to the fact that the majority of bottled water is sold in plastic bottles. It was previously reported that people have concerns about the possible links between the plastic bottle itself and cancer, and also about the detrimental effects of plastic bottles on the environment [55]. Interestingly, this UK study also reported that most participants did not feel that bottled water conferred notable health benefits over tap water. On the other hand, Etale et al. investigated the drivers of increased bottled water consumption and determined convenience as the only contextual predictor of tap water consumption, although for some people, a link with environmental concerns was also observed [56].

In the last part of the consumer study, the participants completed a word-association task, using the brand name Radenska (♥♥♥), which is traditionally labelled with a picture of three hearts (Figure 3). We were particularly interested in whether this brand would be associated with heart health. The participants’ associations with the brand name were arranged into the following categories (Table 5): (1) water—water-related descriptions (e.g., mineral water, (carbonated) water, bubbles etc.), (2) tradition—descriptions related to tradition (e.g., tradition, good brand, Slovenian etc.), (3) Radenska—descriptions directly related to the Radenska brand, (4) health—descriptions related to health, (5) other—answers that did not belong in any of the previous categories. If participants described the logo with words that covered more than one of the aforementioned categories, we only considered the first written association. Altogether, 58% of participants described the brand in a water-related fashion, followed by Radenska- (13%), tradition- (12%) and health-related (9%) descriptions. The word-association task revealed notable differences in participants’ responses. Significant differences were observed between genders (*p* = 0.006) and age groups (*p* = 0.005). Altogether, the Radenska brand was most commonly associated with water, regardless of gender, age or education; differences were observed amongst all the other association categories. It should be mentioned that over the years in Slovenia, the term “radenska” has also become a generic term for mineral water. For example, it is common for “radenska” (as mineral water) to be ordered in a restaurant, even when another brand of mineral water is offered. Other studies also showed that symbols characterize a set of attributes [49] that evolve with the brand’s promotion [57]. Considering that the brand Radenska was promoted as high-quality mineral water, this obviously affected consumer associations. We determined that in most age groups, fewer than 10% of the participants related the Radenska brand with health, with the exception being adults over 55 years of age (15%). These health relationships were mostly referring to general health and not heart health, despite the use of heart images as part of the brand.

While many studies have been conducted in the area of nutritional and health claims on foods, not many have included brand names and trademarks that fall under the same regulations as nutritional and health claims. After 2022 in the EU, all brand names and trademarks that present a nutritional or health claim will need to comply with Regulation EC No 1924/2006, which will have considerable effects on the food supply. Our research will support the recognition of labels on which heart images should be considered as health symbols vs. those where this is not the case. It must be emphasised that each brand name and trademark should be considered individually. It should also be noted that consumers’ perceptions of food are affected not only by elements on the food label but by many other factors. Moreover, consumers’ opinions play an important part in this decision, as brand names and trademarks are formed to increase the sale of the products to them [58], so their understanding is of great importance. Therefore, the marketing of a specific brand can be an important factor affecting consumer behaviour.

Some study limitations should also be mentioned. The study was conducted using an on-line panel, therefore we did not reach people without internet access. Unfortunately, consumer panels in Slovenia are only recruiting Internet users. However, more than 80% of the Slovenian population (16–74 years) is using the Internet [59]. We should also note that while the structure of our sample is comparable with the population based on gender and age, we had a considerably lower proportion of subjects with primary school education (2% vs. 13% in the Slovenian population). A limitation of the study is also that the CA task was undertaken using stimuli–images of food packages, and not with actual foods. This limitation is again related to the on-line conduct of the study. However, in order to make the CA task both realistic and clear, stimuli pictures of foods were accompanied also with the type of product in wording (i.e., stimuli pictures of chocolate were accompanied with worded description ‘chocolate’ (in Slovenian: ‘čokolada’; see Figure 2). This way we ensured that subjects were rating the product with full awareness of the product type.

## 4. Conclusions and Policy Applications

Based on the reported results, we can conclude that the use of images/symbols of the heart is very common on food products. While the use of heart symbols can be used with the intention of relating food products to health [43], such images are also often used in other contexts not related to health. The reported consumer study showed that a heart image as part of the brand name itself—without an additional (specific) health claim—does not cause most consumers to relate it to health. However, consumers tend to relate an image of the heart as part of a brand with health benefits if the said image more directly refers to health, such as if it is accompanied by a worded health claim or designed specifically to imply health benefits. Our research indicates that the tested images of hearts in a brand name, without any other reference to health benefits, should not be considered as a health claim. We should note that in this context the reference to health benefits should be considered in general (taking food marketing into account), not only for the specific food label. Considering that this study was conducted with Slovenian consumers with selected stimuli, care should be taken when applying the study’s results to other designs of brand names and other population groups. Nevertheless, the results of this study highlight that it is not appropriate to declare all use of health images/symbols of the heart on food labels as health claims, the decisions on which should be made on a case-by-case basis.

## Figures and Tables

**Table 1:** Sociodemographic and lifestyle characteristics of participants.

	Number of Participants (%)
**Gender**	
Male	513 (51%)
Female	487 (49%)
**Age**	
18–24	110 (11%)
25–34	195 (20%)
35–44	221 (22%)
45–54	235 (24%)
55–65	239 (24%)
**Education**	
Primary school or less	17 (2%)
Vocabulary school	102 (10%)
High school	379 (38%)
University	502 (50%)
**Diet type**	
Regular (mixed)	932 (93%)
Vegetarian	43 (4%)
Vegan	12 (1%)
Other	13 (1%)
**Regular consumers ^1^ of selected foods**	
Fruits	556 (56%)
Vegetables	567 (57%)
Meats	231 (23%)
Milk and dairy products	361 (36%)
Tap water	899 (90%)
Bottled water	121 (12%)
Soft drinks	141 (14%)
**Total**	1000

Note: ^1^ Consumption frequency was measured on scale 1–7 (1: 3-times per day or more; 2: 1–2 times per day; 3: 4–6 times per week; 4: 2–3 times per week; 5: once per week; 6: 1–3 times per month; 7: never); regular consumers are considered those reporting at least daily consumption (scale 1–2).

**Table 2:** Attributes and attribute levels used in the conjoint analyses task.

Attribute	Attribute Level
Type of product	Soft drink
	Yoghurt
	Chocolate
Brand name	••• Neutral
	♥ One heart
	♥♥♥ Three hearts
Claim	No claim
	General health claim: “*For a healthy heart”*
	Specific health claim: “*For a healthy heart—rich in omega-3, which supports a healthy heart*.”

**Table 3:** Numbers of foods labelled with heart images within different food categories.

Food Category	Number of Food Products	Number of Foods with Heart on FOP	Number of Foods with Heart as Part of the Brand
Beverages	1396	98 (7%)	52 (4%)
Bread and bakery products	1246	152 (12%)	31 (2%)
Cereal and cereal products	1040	107 (10%)	45 (4%)
Confectionary	1141	64 (6%)	5 (0.4%)
Convenience food	549	58 (11%)	41 (7%)
Dairy	1601	123 (8%)	41 (2%)
Edible oils and oil emulsions	304	46 (15%)	30 (10%)
Eggs	38	11 (29%)	0
Fish and fish products	287	5 (2%)	0
Food for specific dietary use	104	53 (51%)	53 (51%)
Fruit and vegetables	1133	38 (3%)	18 (2%)
Meat and meat products	828	135 (16%)	0
Sauces and spreads	653	45 (7%)	19 (3%)
Snack foods	241	28 (12%)	23 (10%)
Sugars, honey and related products	12	4 (33%)	0
Total	10,573	967 (9%)	348 (3%)

FOP: Front-of-package.

**Table 4:** Ratings of health impacts of soft drinks labelled with different constructed brands and claims (N = 1000).

Brand:	♥ One Heart	♥♥♥ Three Hearts	••• Neutral	
Type of Claim:	Mean ± SD ^a^	Mean ± SD ^a^	Mean ± SD ^a^	*p*-Value ^b^
No claim	4.06 ± 1.35	3.98 ± 1.37	3.79 ± 1.32	0.000
General claim	4.40 ± 1.38	4.33 ± 1.34	4.18 ± 1.37	0.000
Specific health claim	4.73 ± 1.47	4.76 ± 1.43	4.66 ± 1.40	0.000

^a^ Measured using a Likert scale of 1 (unfavourable impact) to 7 (favourable impact); SD = standard deviation, ^b^ *p*-values of the differences for the statements between the groups: highly significant differences (*p* < 0.001).

**Table 5:** Percentages of responses within association categories by gender, age and education.

	Number of Participants	Water	Tradition	Radenska	Health	Other	*p*-Value ^a^
**Gender**							
Male	513	55%	12%	14%	8%	11%	0.006
Female	487	61%	12%	11%	10%	6%	
**Age group**							
18–24	110	65%	6%	12%	5%	11%	0.005
25–34	195	59%	13%	16%	6%	6%	
35–44	221	52%	16%	16%	7%	8%	
45–54	235	63%	9%	9%	7%	11%	
55–65	239	55%	13%	10%	15%	6%	
**Education**							
Primary school or less	17	59%	0%	29%	6%	6%	0.128
Vocational school	102	68%	6%	8%	7%	12%	
High school	379	56%	12%	15%	11%	6%	
College or higher	502	58%	14%	11%	8%	9%	
**Total**	1000	58%	12%	13%	9%	8%	

^a^*p*-values of the differences in characteristics between the groups: highly significant differences (*p* < 0.001); very significant differences (*p* < 0.01); significant differences (*p* < 0.05).

## Data Availability

The data presented in this study are available on request from the corresponding author.

## Supplementary Materials

The following are available online at https://www.mdpi.com/2304-8158/10/3/643/s1, Figure S1: Stimuli used for choice-based task with real-life elements, Table S1: Numbers of foods labelled with heart symbol within different food categories and subcategories.
