# Supplementary material for: Heart Images on Food Labels: A Health Claim or Not?
Source: Foods. 2021 Mar 18;10(3):643. doi: 10.3390/foods10030643 (PMC8002900; doi:10.3390/foods10030643)
Supplement: Supplementary file 1 [file foods-10-00643-s001.pdf]

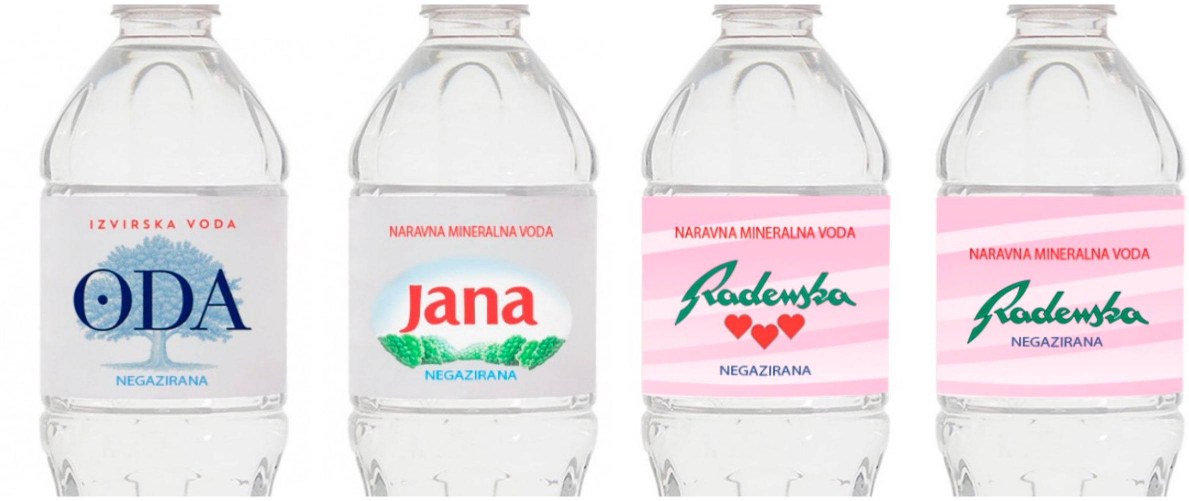

(1) Oda

(2) Jana

(3a) Radenska with image of three hearts

(3b) Radenska without image of three hearts

Figure S1. Stimuli used for choice-based task with real-life elements.

Table S1. Numbers of foods labelled with heart symbol within different food categories and subcategories.

Food category	Number of food products	Number of foods with heart on FOP	Number of foods with heart as part of the brand
**Beverages**	**1396**	**98 (7%)**	**52 (4%)**
Fruit and vegetable juices	301	3 (3%)	0
Soft drinks	348	28 (29%)	4 (8%)
Cordials	92	8 (8%)	7 (13%)
Coffee and tea	560	32 (33%)	23 (44%)
Electrolyte drinks	13	0	0
Waters	82	27 (28%)	18 (35%)
**Bread and bakery products**	**1246**	**152 (12%)**	**31 (2%)**
Bread	154	32 (21%)	0
Biscuits	732	64 (42%)	15 (48%)
Cakes, muffins and pastries	360	56 (37%)	16 (52%)
**Cereal and cereal products**	**1040**	**107 (10%)**	**45 (4%)**
Cereal bars	61	2 (2%)	2 (4%)
Noodles	115	14 (13%)	3 (7%)
Breakfast cereals	297	52 (49%)	30 (67%)
Pasta	325	27 (25%)	9 (20%)
Maize (corn)	16	0	0
Rice	103	5 (5%)	0
Couscous	12	0	0
Unprocessed cereals	111	7 (7%)	1 (2%)
**Confectionary**	**1141**	**64 (6%)**	**5 (0.4%)**
Chocolate and sweets	1075	63 (98%)	5 (100%)
Jelly	6	1 (2%)	0
Chewing gum	60	0	0
**Convenience food**	**549**	**58 (11%)**	**41 (7%)**
Pizza	34	1 (2%)	0
Soup	172	36 (62%)	35 (85%)
Ready meals	297	21 (36%)	6 (15%)
Prepared salads and sandwiches	46	0	0
Other	0	0	0
**Dairy**	**1601**	**123 (8%)**	**41 (2%)**
Cheese	457	18 (15%)	0
Yoghurt products	449	30 (24%)	0
Milk	178	14 (11%)	0
Cream	106	7 (6%)	0
Desserts	185	24 (20%)	18 (44%)
Ice cream and edible ices	226	30 (24%)	23 (56%)
**Edible oils and oil emulsions**	**304**	**46 (15%)**	**30 (10%)**
Butter and margarine	94	7 (15%)	4 (13%)
Cooking oils	210	39 (85%)	26 (87%)
**Eggs**	**38**	**11 (29%)**	**0**
Eggs	38	11 (100%)	0
**Fish and fish products**	**287**	**5 (2%)**	**0**
Canned fish and seafood	165	4 (80%)	0
Chilled fish	44	0	0
Frozen fish	78	1 (20%)	0
**Food for specific dietary use**	**104**	**53 (51%)**	**53 (51%)**
Baby food	104	53 (100%)	53 (100%)
**Fruit and vegetables**	**1133**	**38 (3%)**	**18 (2%)**

Food category	Number of food products	Number of foods with heart on FOP	Number of foods with heart as part of the brand
Vegetables	527	20 (53%)	5 (28%)
Fruit	250	7 (18%)	2 (11%)
Jams and spreads	182	11 (29%)	11 (61%)
Nuts and seeds	174	0	0
**Meat and meat products**	**828**	**135 (16%)**	**0**
Processed meat and derivatives	767	133 (99%)	0
Meat alternatives	61	2 (1%)	0
**Sauces and spreads**	**653**	**45 (7%)**	**19 (3%)**
Sauces	318	16 (36%)	8 (42%)
Mayonnaise/dressings	55	9 (20%)	2 (11%)
Spreads	280	20 (44%)	9 (47%)
**Snack foods**	**241**	**28 (12%)**	**23 (10%)**
Crisps and snacks	241	28 (100%)	23 (100%)
**Sugars, honey and related products**	**12**	**4 (33%)**	**0**
Honey and syrups	12	4 (100%)	0
**TOTAL**	10,573	967 (9%)	348 (3%)
